# Lysophosphatidic Acid Upregulates Recepteur D’origine Nantais Expression and Cell Invasion via Egr-1, AP-1, and NF-κB Signaling in Bladder Carcinoma Cells

**DOI:** 10.3390/ijms21010304

**Published:** 2020-01-01

**Authors:** Pham Ngoc Khoi, Shinan Li, Ung Trong Thuan, Dhiraj Kumar Sah, Taek Won Kang, Thi Thinh Nguyen, Sen Lian, Yong Xia, Young Do Jung

**Affiliations:** 1 Research Institute of Medical Sciences, Chonnam National University Medical School, Gwangju 501-190, Korea; khoicnsh@gmail.com (P.N.K.); shinanli@foxmail.com (S.L.); ungtrongthuan@gmail.com (U.T.T.); dhiraj007sah@gmail.com (D.K.S.); sydad@hanmail.net (T.W.K.); thinhnt1984@gmail.com (T.T.N.); 2 Department of Biochemistry and Molecular Biology, School of Basic Medical Sciences, Southern Medical University, Guangzhou 510515, China; senlian@i.smu.edu.cn; 3 Institute of Precision Medicine, Jining Medical University, Jining 272067, China

**Keywords:** lysophosphatidic acid, recepteur d’origine nantais, bladder cancer, Egr-1, AP-1, NF-κB

## Abstract

Muscle invasive bladder carcinoma is a highly malignant cancer with a high mortality rate, due to its tendency to metastasize. The tyrosine kinase recepteur d’origine nantais (RON) promotes bladder carcinoma metastasis. Lysophosphatidic acid (LPA) is a phospholipid derivative, which acts as a signaling molecule to activate three high affinity G-protein coupled receptors, LPA1, LPA2, and LPA3. This in turn leads to cell proliferation and contributes to oncogenesis. However, little is known about the effects of LPA on invasive bladder cancer (IBC). In this study, we discovered that LPA upregulated RON expression, which in turn promoted cell invasion in bladder cancer T24 cells. As expected, we found that the LPA receptor was essential for the LPA induced increase in RON expression. More interestingly, we discovered that LPA induced RON expression via the MAPK (ERK1/2, JNK1/2), Egr-1, AP-1, and NF-κB signaling axes. These results provide experimental evidence and novel insights regarding bladder malignancy metastasis, which could be helpful for developing new therapeutic strategies for IBC treatment.

## 1. Introduction

Bladder cancer is one of the most common cancers in the world and has a much higher incidence in males and aged individuals [1]. Bladder cancer has been classified into non-invasive bladder cancer (NIBC) and invasive bladder cancer (IBC) by urologists [2]. Although NIBC is responsible for about 70% of bladder cancer cases [3], IBC is more malignant, with a five year survival rate of less than 35% [4,5]. IBC is especially dangerous if it extends from the bladder to the surrounding tissues or spreads to nearby lymph nodes and visceral organs. Therefore, it is particularly important to know the mechanism of bladder cancer cells invasion and to develop therapeutic treatments for IBC. Several molecules promoting cancer cell invasion and metastasis have been identified, of which recepteur d’origine nantais (RON), encoded by the macrophage stimulating protein receptor (*MST1R*) gene, is very important [6]. *MST1R* is related to the *c-MET* receptor tyrosine kinase [7,8]. Upon stimulation by ligands such as macrophage-stimulating protein, RON activates the downstream signaling axis comprising *Src*, *Ras*, and *MAPKs*, which in turn upregulate a number of cell invasion related molecules such as *uPAR* and enhance cancer cell invasiveness [9].

Lysophosphatidic acid (LPA) is a kind of glycerophospholipid, which is an important factor for the development and function of many tissues and organs. LPA presents in many different body fluids such as urine, blood, and saliva [10], playing important roles in the development of the circulatory [11] and nervous [12] systems, functioning of the immune system [13], wound healing [14], and bone metabolism [15]. More interestingly, LPA abnormalities are also involved in tumor progression [16] and autoimmune diseases [17]. LPAs have been known to play a role in a series of tumor development, including stimulation of proliferation, resisting cell death, and evading tumor suppressors by regulating the apoptotic pathways, inducing angiogenesis via upregulation of proangiogenic factors, enabling immortality and activating cell invasion [18]. LPA presumably acts through specific G-protein coupled receptors and is a potent inducer of cell survival, proliferation, and migration. LPA is reported to participate in carcinogenesis and progression of cancers, such as breast cancer [19], ovarian cancer [20], pancreatic cancer [21], and colon cancer [22]. However, there is a lack of research on the role of LPA in bladder cancer.

In this study, IBC patients showed significantly higher RON expression in the urothelium. RT-PCR, Western blotting, and a promoter-luciferase reporter assay showed that LPA induced RON expression in bladder cancer T24 cells. Furthermore, we revealed that LPA induced RON expression through the MAPK (ERK1/2, JNK1/2) Egr-1, AP-1, and NF-κB signaling axes. Our study provided a novel insight into the mechanism of bladder malignancy metastasis, which could be helpful for developing new treatments targeting bladder cancer.

## 2. Results

### 2.1. RON Expression in Bladder Cancer Patients

**Figure 1:**
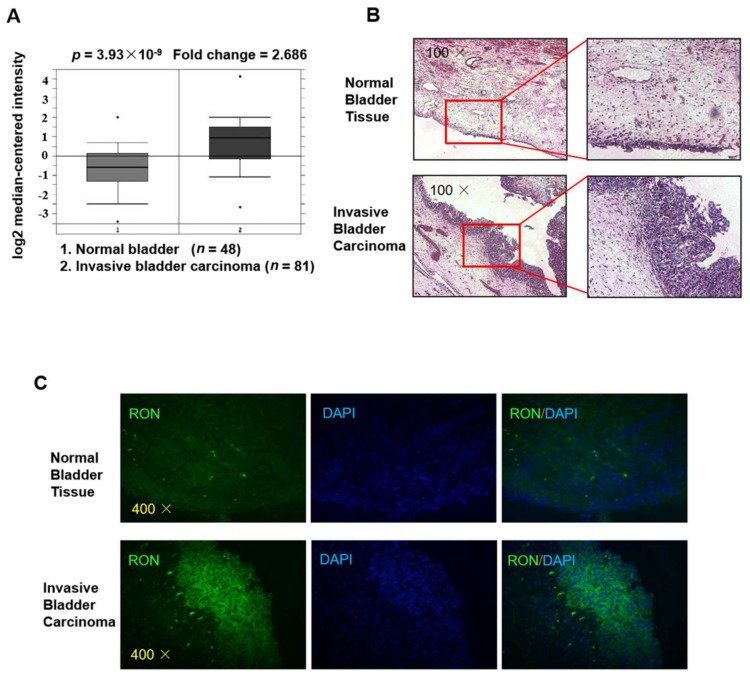
RON expression is higher in invasive bladder cancer than in normal bladder tissue. (**A**) The expression level of RON from a clinical sample database (https://www.oncomine.org). (**B**) H&E staining of normal bladder tissue (upper) and invasive bladder carcinoma (lower) sections. The photographs were taken under a microscope at 100× magnification. (**C**) Immunofluorescence staining was performed to detect RON protein in normal bladder and invasive bladder carcinoma tissue. RON positive cells were stained green, and the nuclei were stained blue. The photographs were taken with a fluorescence microscope at 400× magnification.

As shown in Figure 1A, RON expression in invasive bladder carcinoma patients was significantly higher (2.686 fold, *p* = 3.93 × 10^−9^) than that in healthy controls (clinical data were from Sanchez-Carbayo’s Human Genome U133A Array [23], and the figure was from the Oncomine platform). To verify RON expression in invasive bladder carcinoma, we used immunofluorescence staining for RON in both invasive bladder carcinoma tissue and normal bladder tissue. As shown in Figure 1B,C the RON protein level was significantly higher in the IBC urothelium than in the normal bladder, indicating that RON was closely associated with invasive bladder cancer.

### 2.2. LPA Induced RON Expression in T24 Cells

**Figure 2:**
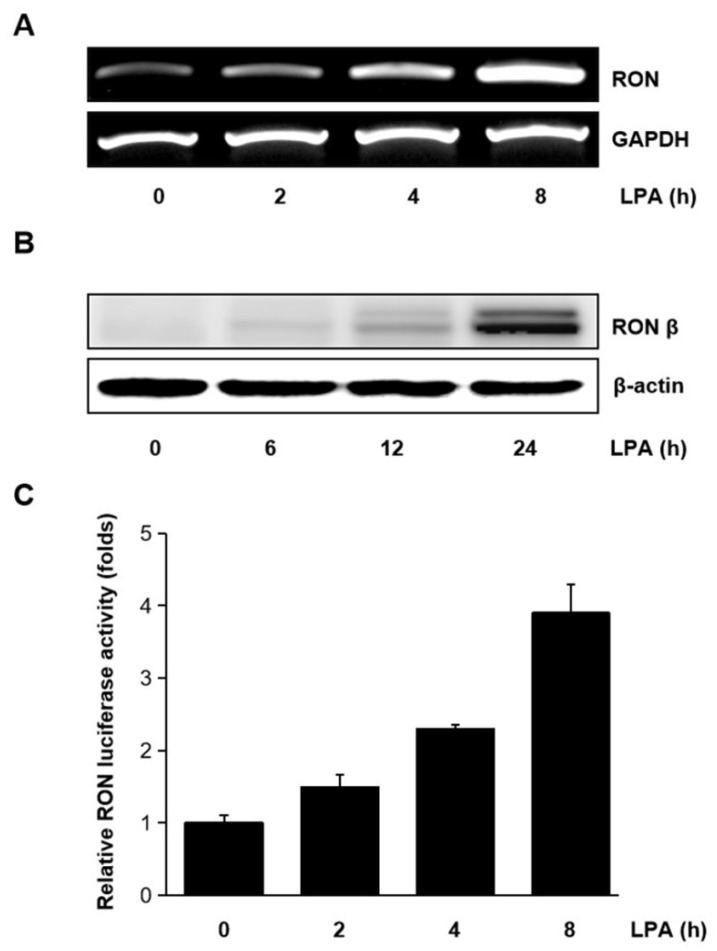
Induction of RON by lysophosphatidic acid (LPA) in T24 bladder cancer cells. (**A**,**B**) RT-PCR and Western blot analyses of the effect of LPA on RON mRNA and protein expression in T24 cells, respectively. Cells were incubated with 5 μM LPA for the indicated durations. (**C**) T24 cells were transiently transfected with the pGL3–RON reporter construct overnight. The transfected cells were incubated with 5 μM LPA for 0–8 h, and luciferase activity was measured with a luminometer. Bars show the mean standard deviation from three measurements.

Human bladder cancer T24 cells were treated with LPA for different periods, and the mRNA level of RON was measured. As shown in Figure 2A,B LPA upregulated the expression of RON mRNA and protein in a time dependent manner. The effect of LPA on the RON transcription was also examined. With the LPA treatment, pGL3-RON transfected T24 cells showed a time dependent increase in luciferase activity (Figure 2C). As shown in Supplementary Figure S1, LPA induced RON expression in a dose dependent manner revealed by Western blot analyses. Supplementary Figure S2 shows that the LPA concentration in the present experiment did not affect T24 cells’ viability.

### 2.3. Role of LPA Receptors in LPA Induced RON Expression

**Figure 3:**
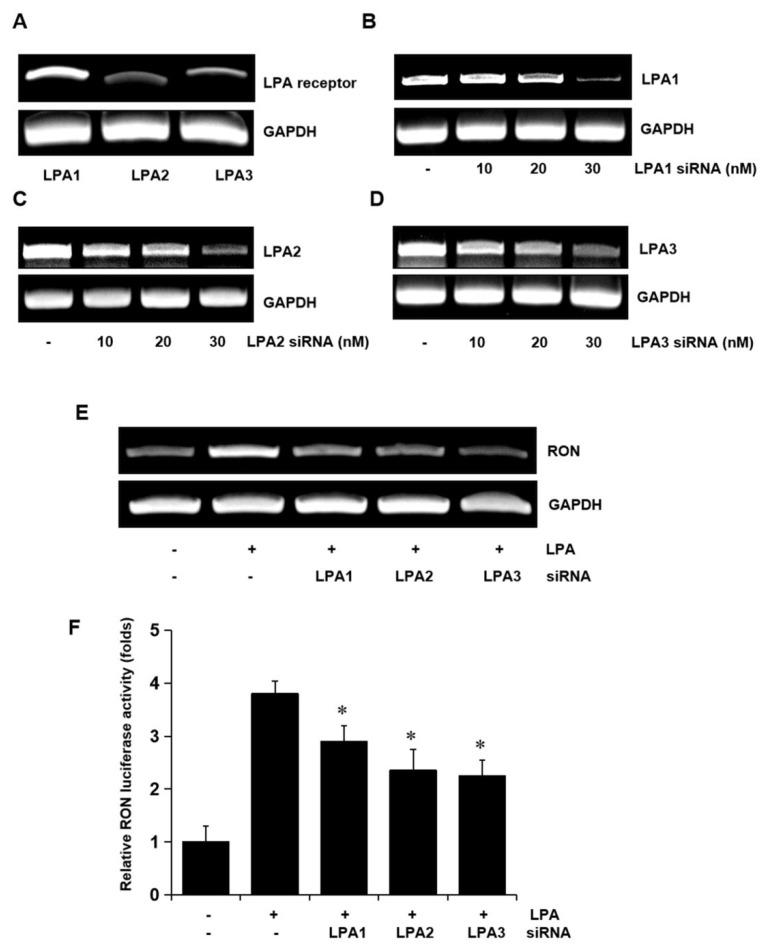
Involvement of LPA receptors in LPA induced RON expression in T24 human bladder cancer cells. (**A**) Total RNA was extracted from T24 cells, and the mRNA levels of LPA1, LPA2, LPA3, and GAPDH were analyzed by RT-PCR. (**B**–**D**) T24 cells were transiently transfected with specific LPA1, LPA2, and LPA3 siRNA oligonucleotides (0–30 nM) for 5 h. After stabilization for 48 h, the amount of LPA1, LPA2, LPA3, and GAPDH mRNAs was determined by RT-PCR. (**E**) T24 cells transfected with 20 nM LPA1, LPA2, and LPA3 siRNA oligonucleotides were incubated with 5 μM LPA for 8 h, and then, RT-PCR was performed. (**F**) T24 cells transfected with specific LPA1, LPA2, and LPA3 siRNAs for 48 h were transiently transfected with a RON promoter reporter construct overnight. The transfected cells were incubated with 5 μM LPA for 8 h, and luciferase activity was measured with a luminometer. Bars show the mean standard deviation from three measurements. * *p* < 0.05 vs. LPA only.

To determine whether the LPA receptors were involved in LPA induced expression of RON, total RNA was extracted from the T24 cells, and LPA1, LPA2, and LPA3 mRNA levels were analyzed by RT-PCR. Three LPA receptors, LPA1, LPA2, and LPA3, were all expressed in T24 cells (Figure 3A). Specific siRNA oligonucleotides were employed to determine which of the LPA receptors were involved in RON induction by LPA. The specific siRNAs for LPA1, LPA2, and LPA3 decreased their corresponding mRNA levels (Figure 3B–D) and partially prevented RON mRNA and promoter activity induced by LPA (Figure 3E,F). As shown in Supplementary Figure S3, double knockdown of two of each among three types of LPA receptor could partially inhibit the LPA induced RON expression, whereas knockdown of all three receptors was almost inhibited in T24 cells, indicating that all three LPA receptors were essential for RON induction by LPA.

### 2.4. Role of Erk-1/2 and JNK MAPK Signals in RON Induction by LPA

**Figure 4:**
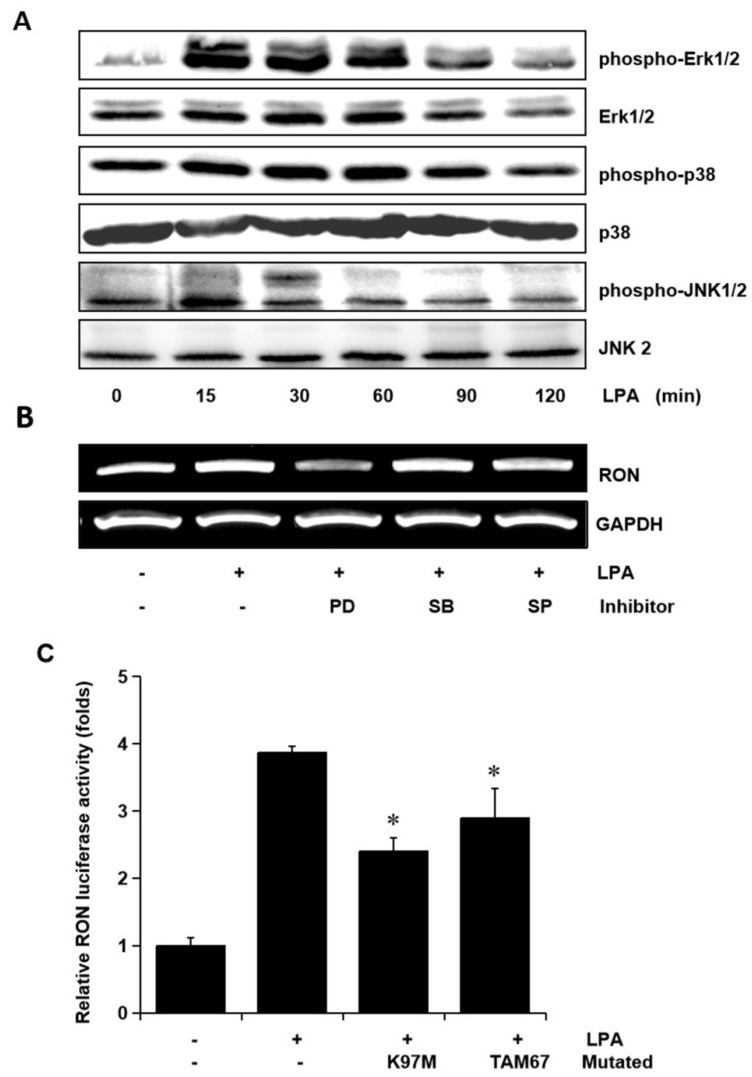
Involvement of Erk-1/2 and JNK in LPA induced RON expression in human bladder cancer T24 cells. (**A**) T24 cells were incubated with 5 μM LPA for various periods, and the levels of phosphorylated Erk-1/2, P38 and JNK MAPK in the cell lysates were determined by Western blot analysis. (**B**) T24 cells, after being pretreated with PD98059 (PD, 50 μM), SB203580 (SB, 10 μM), and SP600125 (SP, 10 μM) for 1 h, were incubated with 5 μM LPA for 8 h. After incubation, the RON mRNA in the cell lysates was determined by RT-PCR analysis. (**C**) T24 cells transfected with Erk-1/2 (K97M) and JNK (TAM67) dominant negative mutants for 48 h were then co-transfected with a RON promoter reporter overnight. After incubation with 5 μM LPA PMA for 8 h, luciferase activity was measured with a luminometer. Bars show the mean standard deviation from three measurements. * *p* < 0.05 vs. LPA only.

To examine whether the MAPK signaling pathways were involved in LPA mediated RON induction, the levels of phosphorylated Erk-1/2, JNK, and P38 MAPK in the T24 cells exposed to LPA for different periods were assessed. The LPA treatment induced an increase in Erk-1/2, JNK, and p38 MAPK phosphorylation, but did not affect their protein levels (Figure 4A). In order to examine the specific signals of MAPKs in RON induction by LPA, T24 cells were pretreated with PD98059 (PD, a MEK inhibitor), SB203580 (SB, a P38 MAPK inhibitor), and SP600125 (SP, a JNK inhibitor) prior to LPA exposure. Figure 4B shows that both the Erk-1/2 inhibitor (PD) and JNK inhibitor (SP) partially blocked RON induction by LPA, whereas the p38 MAPK inhibitor (SB) had no effect. In addition, LPA induced RON promoter activity was also partially inhibited when the expression vector of dominant negative mutants of Erk-1/2 (K97M) or JNK (TAM67) were co-transfected with pGL3-RON into the T24 cells (Figure 4C).

### 2.5. Role of Egr-1 in RON Induction by LPA

**Figure 5:**
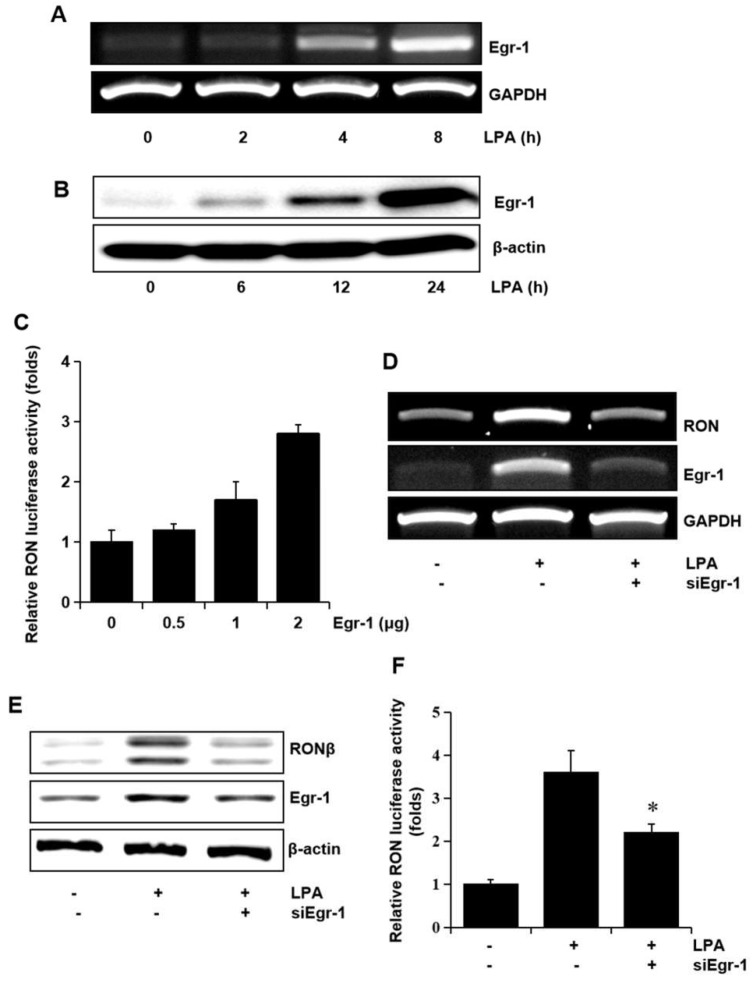
Involvement of the transcription factor Egr-1 in LPA induced RON expression. (**A**,**B**) T24 cells were incubated with 5 μM LPA for indicated durations, and Egr-1 mRNA and protein expression were determined by RT-PCR and Western blot analysis, respectively. (**C**) T24 cells transfected with Egr-1 expression plasmids (0–2 μg) for 48 h were transiently co-transfected with a RON promoter reporter overnight, and luciferase activity was then measured using a luminometer. Bars show the mean standard deviation from three measurements. (**D**,**E**) T24 cells transfected with Egr-1 siRNA oligonucleotides for 48 h were treated with 5 μM LPA for the indicated durations, and RON mRNA and protein expression were determined by RT-PCR and Western blot analysis, respectively. (**F**) T24 cells transfected with Egr-1 siRNA oligonucleotides for 48 h were co-transfected with a RON promoter reporter overnight. After incubation with 5 μM LPA for 8 h, luciferase activity was measured with a luminometer. Bars show the mean standard deviation from three measurements. * *p* < 0.05 vs. LPA only.

Since we have reported that Egr-1 is crucial in RON expression, we assessed whether Egr-1 expression was required for RON induction by LPA. The T24 cells were treated with LPA, and the level of Egr-1 mRNA and the protein level were determined. As shown in Figure 5A,B LPA upregulated the expression of Egr-1 mRNA and protein in a time dependent manner. In addition, the expression vector of Egr-1 increased RON promoter activity in a dose dependent manner (Figure 5C). To confirm the critical role of Egr-1 in LPA induced RON expression in T24 cells, an RNA interference approach was employed. RT-PCR and Western blot analyses showed that transient expression of an siRNA targeting Egr-1 resulted in a decreased level of RON and Egr-1 expression (Figure 5D,E). LPA induced RON promoter activity was decreased in T24 cells transfected with Egr-1 siRNA determined by the promoter study (Figure 5F).

### 2.6. Role of AP-1 and NF-κB in RON Induction by LPA

**Figure 6:**
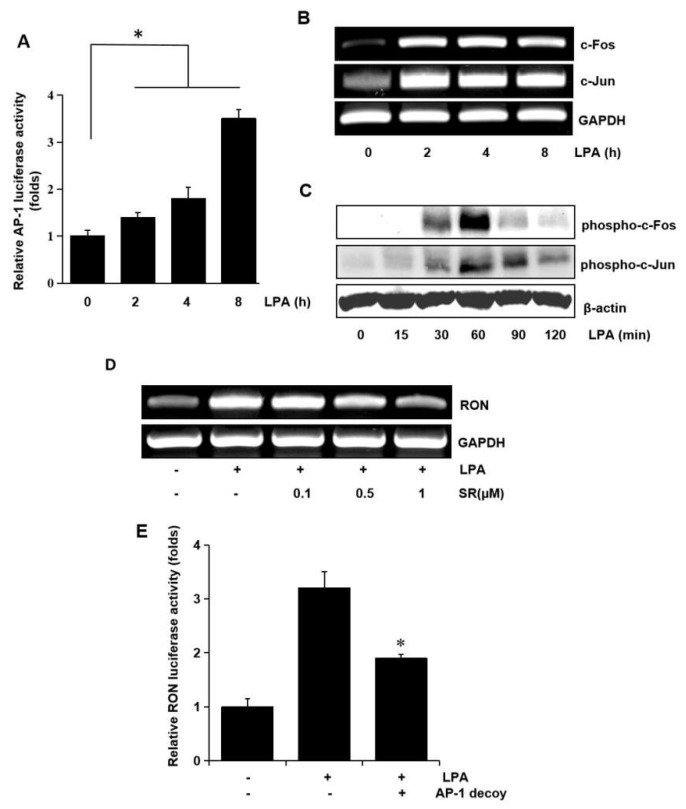
Involvement of the AP-1 transcription factors in LPA induced RON expression. (**A**) Cells were transiently transfected with the pAP-1 luciferase reporter construct and then incubated with 5 μM LPA for 0–8 h. After incubation, the cells were lysed, and luciferase activity was measured. Bars show the mean standard deviation from three measurements. (**B**) T24 cells were incubated with 5 μM LPA for 0–8 h, and the mRNA levels of c-fos and c-jun were determined by RT-PCR. (**C**) T24 cells were incubated with 5 μM LPA for 0–120 min, and the levels of c-fos and c-jun phosphorylation were determined by Western blot analysis. (**D**) T24 cells pretreated with SR11032 (SR, AP-1 inhibitor, 0–1 μM) were incubated with 5 μM LPA for 8 h, and RON mRNA levels were determined by RT-PCR. (**E**) T24 cells were transfected with an AP-1 decoy for 48 h and co-transfected with a RON promoter reporter overnight. After incubation with 5 μM LPA for 8 h, luciferase activity was measured with a luminometer. Bars show the mean standard deviation from three measurements. * *p* < 0.05 vs. LPA only.

In a previous study, we reported potential roles for the transcription factors, AP-1 and NF-*κ*B, in RON expression [9]. Therefore, in this study, we examined the role of AP-1 and NF-κB in RON expression by LPA in bladder cancer T24 cells. AP-1 dependent transcription studies have shown that LPA activates AP-1 in a dose dependent manner (Figure 6A). Since c-fos and c-jun are elements of AP-1, we examined the effect of LPA on c-fos and c-jun in T24 cells. As shown in Figure 6B,C LPA induced not only c-fos and c-jun expression at the transcriptional level, but also their phosphorylation, as determined by RT-PCR and Western blotting, respectively. The involvement of AP-1 in LPA induced RON expression was examined by pretreating T24 cells with SR11302 (SR), which is an AP-1 inhibitor. RT-PCR and promoter studies showed that treatment with SR abrogated LPA induced RON expression (Figure 6D,E).

**Figure 7:**
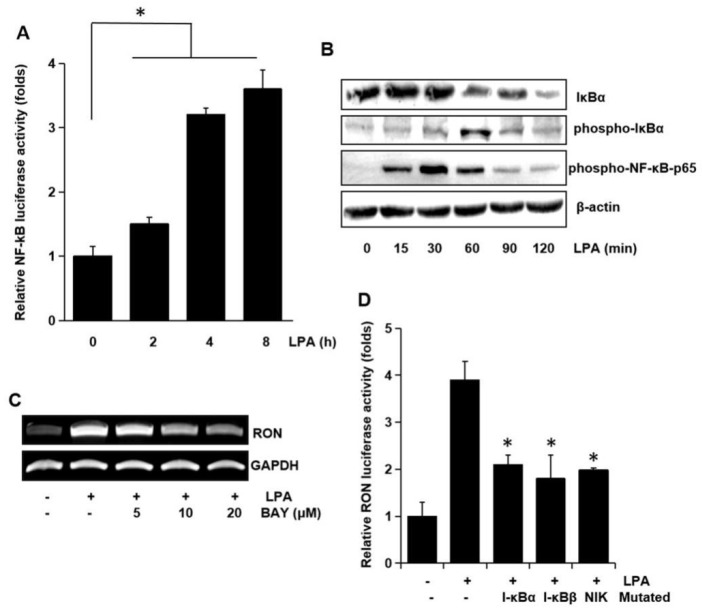
Involvement of the NF-κB transcription factor in LPA induced RON expression. (**A**) Cells were transiently transfected with the pNF-κB luciferase reporter construct and then incubated with 5 μM LPA for 0–8 h. After incubation, the cells were lysed, and luciferase activity was measured. Bars show the mean standard deviation from three measurements. (**B**) T24 cells were incubated with 5 μM LPA for 0–120 min, and the protein level of IκBa and level of IκBa and NF-κB-p65 phosphorylation were determined by Western blot analysis. (**C**) T24 cells were pretreated with BAY11-7082 (BAY, an NF-κB inhibitor, 0–20 μM) were incubated with 5 μM LPA for 8 h, and RON mRNA levels were determined by RT-PCR. (**D**) T24 cells transfected with I-κBα, I-κBβ, and NIK dominant negative mutants were co-transfected with a RON promoter reporter overnight. After incubation with 5 μM LPA for 8 h, luciferase activity was measured with a luminometer. Bars show the mean standard deviation from three measurements. * *p* < 0.05 vs. LPA only.

To examine the involvement of the transcription factor NF-κB on RON expression, NF-κB dependent transcription activities were assayed. As shown in Figure 7A LPA activated NF-κB dependent promoter activity in a time dependent manner. To clarify the underlying mechanisms in LPA induced activation of NF-κB transcriptional activity, we assessed the phosphorylation of NF-κB components by Western blotting analyses. LPA was found to change the level of IκBα and phosphorylation of IκBα and NF-κB p65 in a time dependent manner (Figure 7B). BAY-11-7082 (BAY), which is an NF-κB inhibitor, inhibited the RON mRNA induction by LPA (Figure 7C). The involvement of NF-κB in RON induction by LPA was confirmed by co-transfection into T24 cells with dominant negative forms of NF-κB related molecules and a RON reporter. Figure 7D shows that the dominant negative forms of IκBα, IκBβ, or NIK inhibited the LPA induced RON promoter activity. The above suggested that AP-1 and NF-κB played a major role in LPA induced RON expression.

### 2.7. Effect of LPA on T24 Cell Invasion

**Figure 8:**
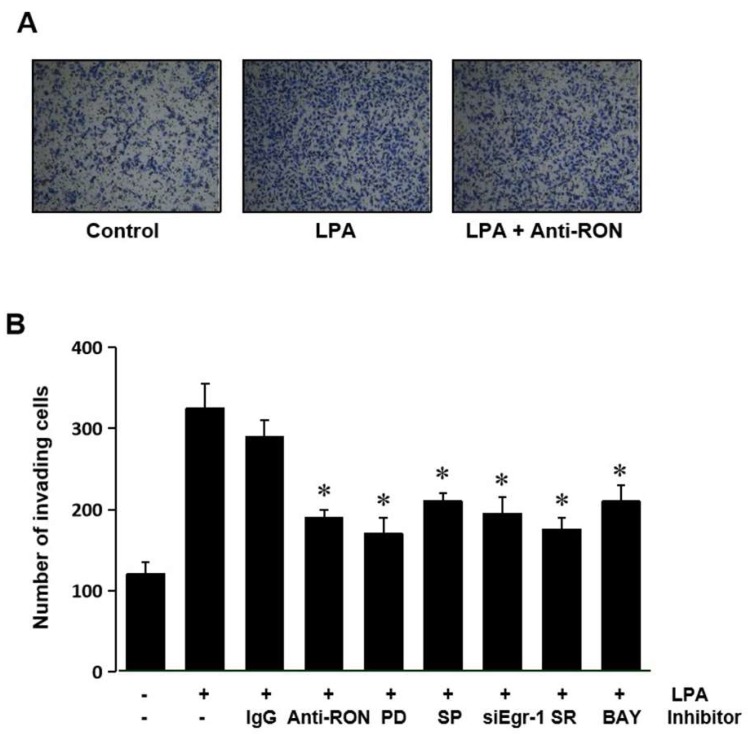
LPA effect on T24 cell invasiveness. (**A**) T24 cells (10^5^) were seeded onto Matrigel coated membranes of a chamber with 5 μM LPA for 24 h, in the presence or absence of anti-RON antibody (200 ng/mL). After incubation, the cells that had invaded the lower chambers were fixed with 100% methanol and stained with a Diff-Quick stain kit. Images of the invading T24 cells were obtained using a phase contrast light microscope. (**B**) T24 cells (10^5^) were seeded onto Matrigel coated membranes of a chamber with 5 μM LPA for 24 h, in the presence or absence of non-specific IgG (200 ng/mL), anti-uPA antibody (200 ng/mL), anti-uPAR antibody (200 ng/mL), siEgr-1 (20 nM, cells transfected with Egr-1 siRNA oligonucleotides for 48 h before LPA treatment), PD98059 (PD, 50 μM), SB203580 (SB, 10 μM), SP600125 (SP, 10 μM), SR11032 (SR, 1 μM), and BAY11-7082 (BAY, 10 μM). After incubation, the cells that had invaded the lower chambers were counted. The bars show the mean and SD from three measurements. * *p* < 0.05 vs. LPA only.

Expression of RON has been suggested to be essential for the invasive phenotype of cancer cells. The role of RON induced by LPA in T24 cells’ invasion was assessed by culturing the cells with an antibody specific for RON in a modified Boyden invasion chamber. Figure 8A shows that LPA could increase the cell invasiveness; however, the RON antibody can weaken the LPA induced cell invasion. These results indicate that induction of RON by LPA plays an important role in the invasiveness of bladder cancer cells. To confirm that MAPK (Erk-1/2 and JNK), Egr-1, AP-1, and NF-kB are involved in LPA induced T24 cell invasiveness, cells were pretreated with various signaling inhibitors PD98059 (PD), SP600125 (SP) siEgr-1, SR11302 (SR), and BAY11-7082 (BAY) prior to LPA treatment. Figure 8B shows that all the inhibitors used blocked LPA induced matrigel invasiveness. These results suggest that MAPK (Erk-1/2 and JNK) and transcription factors (Egr-1, AP-1, and NF-κB) activated by LPA upregulated RON, in turn leading to an increase in bladder cancer cell invasiveness.

## 3. Discussion

Invasiveness and metastasis account for most of the deaths from urothelial bladder cancer because: (1) Invasive bladder carcinoma cells tend to grow toward the mucosal and muscular layers, making it difficult to clear them completely by surgery [24]; (2) When invasive bladder carcinomas invade the mucosal or muscular layer and come in contact with a lymph node or blood vessel, the cancer cells can easily migrate to and metastasize other tissues or organs [25,26]; (3) Invasive bladder carcinoma does not usually develop from non-invasive bladder carcinoma, but has its own bio-marker signatures such as Krt5, Krt14, and CD44 [27] and has a poorer prognosis [24]. Before invading into the mucosal or muscular layers, cancer cells need to break through the extracellular barriers that separate different tissues [28]. During cancer cells’ invasion, degradation of the extracellular matrix requires high levels of hydrolases, such as matrix metalloproteinases (MMPs) [29], urokinase-type plasminogen activator (uPA), and its receptor (uPAR) [30]. In our previous study, we found that stimulation of RON by macrophage stimulating protein (MSP) upregulated uPAR expression, which enhanced gastric cancer cell invasiveness [9]. In this study, we discovered that LPA was able to induce higher RON expression, which in turn promoted bladder cancer T24 cells’ invasiveness. LPA is widely present in human bodily fluids including urine [10]. It was discovered that LPA interacted with specific G-protein coupled receptors (LPA receptors) on the cell surface and in turn activated LPA receptors. The activated LPA receptors could initiate a cascade amplifying multiple signaling pathways, including RAS controlled cell cycle progression and RHO/RAC signaling mediated cell migration and invasion [31]. In our present study, we demonstrated that LPA induced RON expression in the bladder cancer cell line T24 via interaction with LPA receptors, especially LPA2 and LPA3. Consistent with previous reports that the LPA-LPAR binding can activate downstream signaling cascades including MAPK signaling pathways [12], we found that LPA could activate ERK1/2 and JNK1/2 rapidly, but could not activate the p38 MAPK signal. Moreover, our study demonstrated that ERK1/2 and JNK1/2 were indispensable for LPA induced RON expression.

Egr-1 (early growth response protein 1, also known as Zif268 or NGFI-A), a protein encoded by the *EGR1* gene in humans, is localized in nucleus and functions as a transcriptional regulator [32]. In our previous study, we found that Egr-1 was the essential transcription factor for RON expression [33]. In this study, it was discovered that LPA elevated Egr-1 protein levels, which were positively correlated to RON expression (Figure 5). Our present paper was the first to report on LPA induction of Egr-1. Other researchers have also reported that LPA induces Egr-1 expression via the LPA cognate receptor (LPA receptor 1) dependent and PKCδ mediated pathways in vascular smooth muscle cells [34]. It was also discovered that LPA elevated Egr-1 levels via LPA receptor mediated MAPK activation in human osteosarcoma MG-63 cells [35]. AP-1 (activator protein 1), an important transcription factor, regulates gene expression in response to various stimuli, including cytokines, growth factors, infections, and stress [36]. We previously discovered that AP-1 was critical for RON expression in gastric cancer cells [9]. Recently, Zhang et al. also demonstrated that AP-1′s subunit c-Fos would be a suitable target for suppressing proliferation and viability in bladder cancer T24 cells [37]. In the current study, we found that LPA induced RON via upregulation of the AP-1′s subunit components c-jun and c-fos, as well as enhancing phosphorylation of c-Jun and c-Fos in bladder cancers (Figure 6). Our previous and present studies found that besides Egr-1 and AP-1, NF-κB was also critical for RON expression. NF-κB, short for nuclear factor kappa light chain enhancer of activated B cells, is composed of a group of inducible transcription factors that have essential functions in human cells [38]. NF-κB activity is regulated by a family of proteins known as IKKs and IκBs. When inactivated, NF-κB proteins are complexed with IκBs and localized to the cytoplasm. The NF-κB complex can be activated by various inflammatory molecules, cytokines, and several carcinogens. Once an NF-κB complex is activated, IKKs can phosphorylate the IκBs, and the phosphorylated IκBs are ubiquitinated and marked for degradation by proteases. After IκB degradation, the activated NF-κB p65 subunit is released, which is translocated into the nucleus with p50, upon which it activates transcription of its target genes [39,40]. The importance of NF-κB in promoting cancer malignancy has been reveled in various cancers. Recently, it was reported that NF-κB plays a critical role in regulation of urothelial bladder cancer cell proliferation related molecules’ expression [41] and promotes bladder cancer cell migration via the RhoGDIα pathway [42]. In this study, we discovered that LPA activated NF-κB by inducing NF-κB p65 phosphorylation, which is essentially required for NF-κB translocation into the nucleus. If NF-κB was silenced, the induction of RON by LPA was markedly reduced, which indicated that NF-κB was indispensable for LPA induced RON expression (Figure 7). To illustrate the whole mechanism visually, we constructed a diagram (shown as the Graphical Abstract) based on what we discovered in this study to illustrate the cell signaling pathways by which LPA induced RON expression in bladder cancer T24 cells. Since LPA was established as a target for cancer and the concept of targeting LPA needed to be moved to clinical cancer treatment [18], our present findings provided experimental evidence that could be helpful for developing new therapeutic strategies for bladder cancer therapy targeting LPA. Based on what we have found in this study, that LPA increased RON expression in an LPA receptor dependent manner, suppressing the LPA level or blocking LPA and the LPA receptor would be effective ways to suppress RON expression and, in turn, inhibit cell invasion.

## 4. Materials and Methods

### 4.1. Clinical Data Analysis and Specimen Collection

RON expression data were collected from the Sanchez-Carbayo’s dataset [23] via the Oncomine platform (www.oncomine.org). De-identified bladder tumor specimens were obtained from Chonnam National University Hwasun Hospital with institutional review board (IRB) approval with the code of CNUH-06-070 (13 December 2006).

### 4.2. H&E Staining

Five micrometer thick sections were cut from formalin fixed paraffin embedded blocks for H&E and immunohistochemistry (IHC) staining. The sections were first immersed in xylene and then soaked successively in 100% ethanol, 90% ethanol, and 70% ethanol. After washing for 15 min with flowing water, the sections were incubated in hematoxylin solution for 5 min and washed with flowing water for 15 min. Subsequently, eosin staining was then carried out by soaking in eosin solution for 1 min and then successively washing with 95% ethanol, 100% ethanol, and 100% xylene. Finally, the sections were mounted and imaged under a microscope.

### 4.3. Immunofluorescencent Staining

After being dewaxed by successive immersion in 100% xylene, 100% ethanol, 90% ethanol, 70% ethanol, and running water, the sections were heated in a microwave to unmask and repair the antigen for immunofluorescence staining. Nonspecific binding sites were blocked with 1% BSA in PBS. The primary antibody, anti-RON (1:200, Novus Biologicals, CO, USA), was added to the samples and incubated for 14 h at 4 °C. The sections were then incubated with the secondary antibody and Alexa-488 dye (1:500, Invitrogen, Eugene, OR, USA). After being mounted with an aqua mount, the slides were imaged under a microscope (AxioPlan 2; Zeiss, Germany).

### 4.4. Cell Culture

Bladder cancer T24 cells were obtained from the ATCC (Manassas, VA, USA) and cultured in DMEM (Logan, UT, USA) mixed with 0.6% penicillin-streptomycin and 10% FBS in 5% CO_2_ at 37 °C. When the confluence reached 50–60%, the cells were treated with different concentrations of LPA (Sigma-Aldrich, St. Louis, MO, USA). To investigate the effects of LPA on RON expression in T24 cells, the cells were collected at various times, and the RON expression levels were assessed by reverse transcription polymerase chain reaction (RT-PCR) and Western blot analyses. The roles of specific signal transduction pathways in LPA induced RON expression were determined by pretreatment of T24 cells with the specific MAPK/Erk kinase (MEK) inhibitor PD98059 (NEB, Beverly, MA), p38 MAPK inhibitor SB203580 (Calbiochem, La Jolla, CA, USA), JNK inhibitor SP600125 (Calbiochem, La Jolla, CA, USA), NF-κB inhibitor BAY11-7082 (Calbiochem, La Jolla, CA, USA), and AP-1 inhibitor SR11302 (Tocris Bioscience, Ellisville, MO, USA) for 1 h prior to exposure to LPA.

### 4.5. Reverse Transcription-Polymerase Chain Reaction

Total RNA was obtained from the T24 cells with TRIzol reagent (Invitrogen, Carlsbad, CA, USA). One microgram of the total RNA was used for first strand cDNA synthesis using random primers and Superscript reverse transcriptase. The cDNA was subjected to PCR amplification with the primer sets for GAPDH, LPA1, LPA2, LPA3, RON, and Egr-1. The specific primer sequences were as follows: GAPDH sense, 5′-TTG TTG CCA TCA ATG ACC CC-3′, and GAPDH antisense, 5′-TGA CAA AGT GGT CGT TGA GG-3′ (836 bp); LPA1 sense, 5′0-AAT CGA GAG GCA CAT TAC GG-3′, and LPA1 antisense, 5′-TGT GGA CAG CAC ACG TCT AG-3′ (432 bp); LPA2 sense, 5′-CAT CAT GCT TCC CGA GAA CG-3′, and LPA2 antisense, 5′-GGG CTT ACC AAG GAT ACG CAG-3′ (352 bp); LPA3 sense, 5′-AGG ATG CGG GTC CAT AGC AA-3′, and LPA3 antisense, 5′-GAT GAT GGG GTT CAC GAC GG-3′ (481 bp); RON sense, 5′-ACG GCT TAG CGC CAC TG AGC-3′, and RON antisense, 5′-CAT GTG TGC CAC TGT GAC GT-3′ (550 bp); Egr-1 sense, 5′-CAG TGG CCT AGT GAG CAT GA-3′, and Egr-1 antisense, 5′-CCG CAA GTG GAT CTT GGT AT-3′ (767 bp). The PCR conditions were as follows: denaturation at 94 °C for 30 s, annealing at 58 °C for 40 s and extension at 72 °C for 40 s, repeat 30 cycles, using a Thermal Cycler (T100, Bio-Rad, USA, Hercules, CA, USA).

### 4.6. Western Blot Analysis

Cells were suspended in ice-cold RIPA buffer, and cell lysate proteins (100 μg) were electrophoresed on SDS polyacrylamide gel and then transferred to a nitrocellulose membrane. The blots were blocked for 1 h at room temperature in a blocking solution (5% non-fat dry milk in Tris buffered saline containing 0.05% Tween-20; TBST), and then immersed in primary antibody (4 °C, overnight, shaking). Immunoreactive signals were generated in a luminescence detection system (Amersham, Franklin Lakes, NJ, USA) using horseradish peroxidase labeled secondary antibody (Cell Signaling Technology, Danvers, MA, USA). The primary antibodies used were as follows: anti-RONβ was from Santa Cruz Biotechnology, and anti-phosphospecific Erk-1/2, anti-phosphospecific JNK anti-phosphospecific p38 MAPK, anti-Egr-1, anti-phosphorylated c-jun, anti-phosphorylated c-fos, anti-IκBα, anti-phosphorylated IκBα (Ser32), and anti-phosphorylated NF-κB p65 antibodies were purchased from Cell Signaling Technology. To measure the loading quantity of samples, the blotted membrane was stripped with 62.5 mM Tris−HCl (pH 7.4) containing 2% SDS and 100 mM 2-mercaptoethanol, followed by hybridization with β-actin antibody (Santa Cruz, Dallas, Texas, USA) or antibodies against total Erk-1/2, JNK and p38MAPK (Cell Signaling Technology, Danvers, MA, USA).

### 4.7. RON Promoter Assay

To investigate the transcriptional regulation of RON, a RON promoter–luciferase reporter construct (pGL3–RON) was employed: when cells’ confluence reached 60–70%, the pGL3-RON promoter–luciferase construct was transfected into T24 cells with FuGENE 6 (Promega, Madison, WI, USA) reagent. The cells were also transfected with the pRL-TK plasmid containing Renilla luciferase as an internal control. The levels of Renilla and firefly luciferase activities were measured with the Dual-Glo Luciferase Assay System (Promega, Madison, WI, USA). The firefly luciferase activities were normalized to Renilla luciferase activity. After transfection overnight and treatment with LPA for 8 h, the T24 cells were detached and lysed with lysis reagent (Promega, Madison, WI, USA). The luciferase activities in the cell lysate supernatant were then measured using a luminometer. The effects of signaling inhibitors on RON promoter activities were examined by pretreatment of cells with the inhibitors for 1 h prior to the addition of LPA. The co-transfection studies were carried out in the presence or absence of AP-1 decoy oligodeoxynucleotides (ODN), IκBα, IκBβ, nuclear factor-κB-inducting kinase (NIK), MEK-1 (pMCL-K97M), or c-Jun (TAM67). The phosphorothioate double stranded ODNs having a sequence complementary (AP-1 decoy ODNs) to the AP-1 binding site (5′-CACTCAGAAGTCACTTC-3′ and 3′-GAAGTGACTTCTGAGCTG-5′) were prepared. The plasmids respectively encoding the inactive c-Jun (TAM67) and MEK-1 (pMCL-K97M) were kindly provided by Dr. Ahn, N.G. [43] and Dr. Birrer, M.J. [44], respectively. The NIK mutants and dominant negative IκBα and IκBβ were generous gifts from Dr. Green, W.C. [45] and Dr. Ballard, D.W. [46], respectively. The critical role of Egr-1 in the expression of RON was determined by co-transfecting cells with pGL3–RON and an Egr-1 expressing plasmids containing the full-length cDNA coding Egr-1 (kindly provided by Dr. Young Han Lee, Konkuk University, Seoul, Korea).

### 4.8. Gene Silencing by siRNA

Specific duplex small interfering RNAs were used to silence the human LPA1, LPA2, LPA3, and human Egr-1 (sc-29303; Santa Cruz Biotechnology, Santa Cruz, CA, USA). The siRNA sequences for LPA1, LPA2, and LPA3 were designed as follows. LPA1; 5′-GAAAUGAGCGCCACCUUUATT-3′, LPA2; 5′-GGUCAAUGCUGCUGUGUACTT-3′, LPA3; 5′-CAGCAGGAGUUACCUUGUUTT-3′. Briefly, two separate tubes for each transfection reaction containing 10–30 nM siRNA oligonucleotides and 2 μL of Lipofectamine RNAiMAX (Invitrogen, Carlsbad, CA) were respectively mixed with 100 μL of the serum-free medium Opti-MEM (Hyclone, Logan, UT, USA) and incubated for 5 min at room temperature. The contents of the two separate tubes were combined and incubated to form siRNA–lipofectamine complexes for 30 min at room temperature. Eight-hundred microliters of T24 cells cultured in serum-free medium were added to the siRNA–lipofectamine mix, plated in a six well tissue culture dish, and incubated at 37 °C and 5% CO_2_ for 5 h. The transiently transfected cells were then stabilized in normal growth medium for 48 h.

### 4.9. AP-1 and NF-κB Promoter Driven Reporter Assay

The plasmids of the AP-1 and NF-κB promoter reporter were obtained from Clontech (Palo Alto, CA, USA). The T24 cells with a confluence of 60–70% were transiently transfected with the AP-1 or NF-κB promoter driven reporter plasmids using a FuGENE 6 transfection kit according to the protocols. The AP-1 or NF-κB promoter-driven reporter plasmid transfected cells were treated with 5 μM LPA for 4 h, and then luciferase activities were measured.

### 4.10. Cells Invasiveness Assay

Membranes containing micropores (Neuro Probe) were used for the cells’ invasion assay in a 10 well chemotaxis chamber. T24 or siEgr-1 transfected cells in a 200 μL medium were placed in the upper chamber with LPA, an MEK inhibitor (PD, PD98059), JNK inhibitor (SP, SP600125), AP-1 inhibitor (SR, SR11032), NF-κB inhibitor (BAY, BAY11-7082), or the RON antibody. The lower chamber was filled with DMEM containing 10% FBS to act as a chemo attractant. After 24 h of incubation, the non-invading cells on the upper surface of the membrane were swabbed away, and the invaded cells were stained using a Diff-Quick kit (Becton-Dickinson, Franklin Lakes, NJ, USA) on the lower surface. After being rinsed twice with distilled water, the invaded cells were counted under a microscope.

### 4.11. Cell Viability Assay

Bladder cancer T24 cells were cultured in a 96 well plate for 24 h. Different concentrations of LPA were added to treat the cells for 8 h. Then, the LDH-Cytox Assay Kit was employed to detect the cell viability following the manufacturer’s instructions.

### 4.12. Statistical Analysis

All values in figures are expressed as the mean ± the standard deviation (SD) and represent the mean of three independent experiments. The data between groups were compared with a *t*-test, and *p* < 0.05 was considered statistically significant.

## 5. Conclusions

Our study identified that LPA stimulated the LPA receptors LPA1, LPA2, and LPA3, activating the downstream ERK1/2 and JNK1/2 signaling, which in turn upregulated Egr-1 expression or c-Jun, c-Fos, and NF-κB p65 phosphorylation and nucleus translocation. LPA activated AP-1, NF-κB, and Egr-1, upregulated RON expression, and thus, promoted T24 bladder cancer cells’ invasiveness. Although we revealed one mechanism by which LPA induced RON expression, we cannot exclude other possible mechanisms. Our study provided novel insights into the mechanism of bladder cancer cells’ invasion, which could be helpful for developing new therapeutic strategies targeting LPA induced RON expression.

## Supplementary Materials

Supplementary Materials can be found at https://www.mdpi.com/1422-0067/21/1/304/s1.
