# Supplementary material for: Lysophosphatidic Acid Upregulates Recepteur D’origine Nantais Expression and Cell Invasion via Egr-1, AP-1, and NF-κB Signaling in Bladder Carcinoma Cells
Source: Int J Mol Sci. 2020 Jan 1;21(1):304. doi: 10.3390/ijms21010304 (PMC6981588; doi:10.3390/ijms21010304)
Supplement: Supplementary file 1 [file ijms-21-00304-s001.zip › Supplementary Figures.pdf]

Supplementary Figures

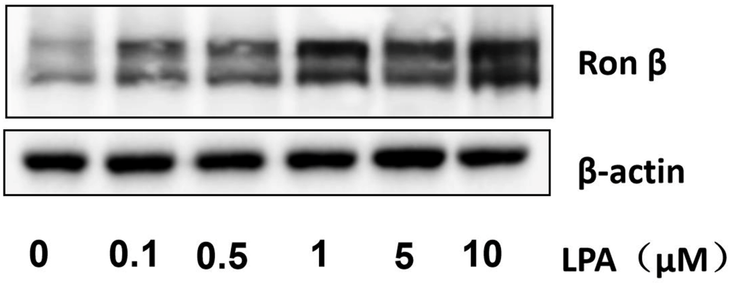

Figure S1. LPA induced RON expression dose-dependently. Western blot was performed to detect the effect of different concentration of LPA on RON protein expression in T24 cells.

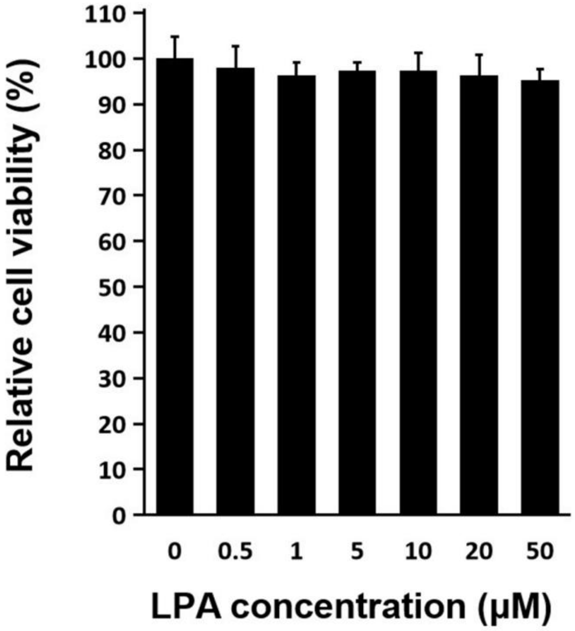

Figure S2. The effect of LPA on T24 human bladder cancer cells viability. T24 cells were cultured into 96-well plate for 24 h. And different concentration (0, 0.5, 1, 5, 10, 20 and 50 μM) of LPA were added to treat the cells for 8 h. Then the LDH-Cytox Assay Kit was employed to detect the cell viability followed the manufacturer's instruction.

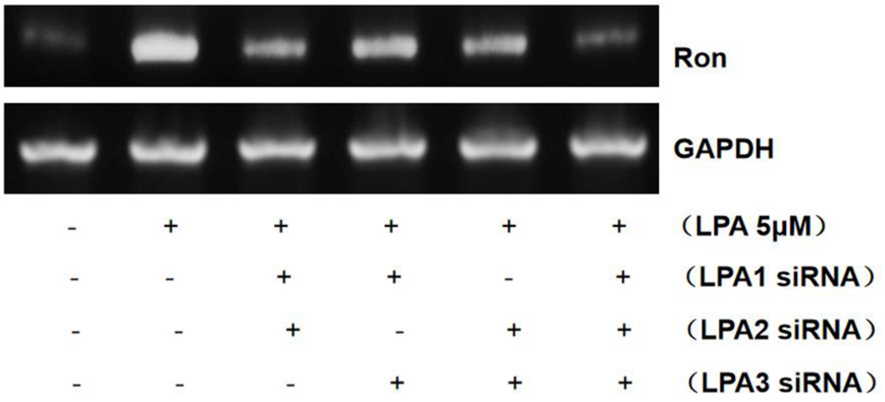

Figure S3. Involvement of LPA receptors in LPA-induced RON expression in T24 human bladder cancer cells. T24 cells were transiently transfected with specific LPA1/LPA2, PLA2/LPA3, PLA1/PLA3 or LPA1/LPA2/LPA3 siRNA oligonucleotides (20 mM) for 5 h. After stabilization for 48 h, the transfected T24 cells were incubated with 5 μM LPA for 8 h and then RT-PCR was performed to examine the mRNA of RON and GAPDH.
