# Supplementary material for: Lysophosphatidic Acid Upregulates Recepteur D’origine Nantais Expression and Cell Invasion via Egr-1, AP-1, and NF-κB Signaling in Bladder Carcinoma Cells
Source: Int J Mol Sci. 2020 Jan 1;21(1):304. doi: 10.3390/ijms21010304 (PMC6981588; doi:10.3390/ijms21010304)
Supplement: Supplementary file 1 [file ijms-21-00304-s001.zip › Western Blot Figures.pptx]

## Slide 1
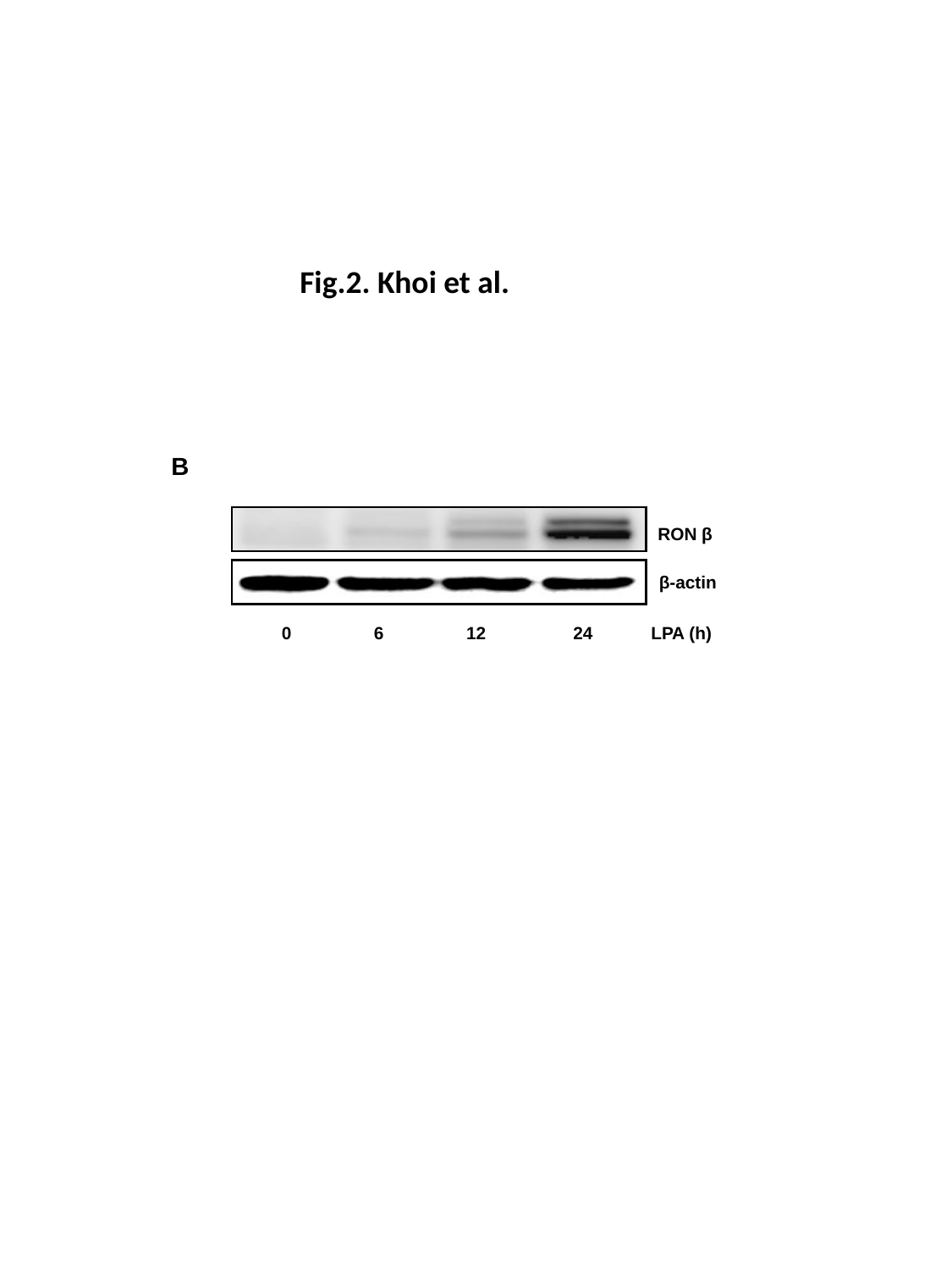

Fig.2. Khoi et al.
B
RON β
β-actin
 0 6 12 24 LPA (h)

## Slide 2
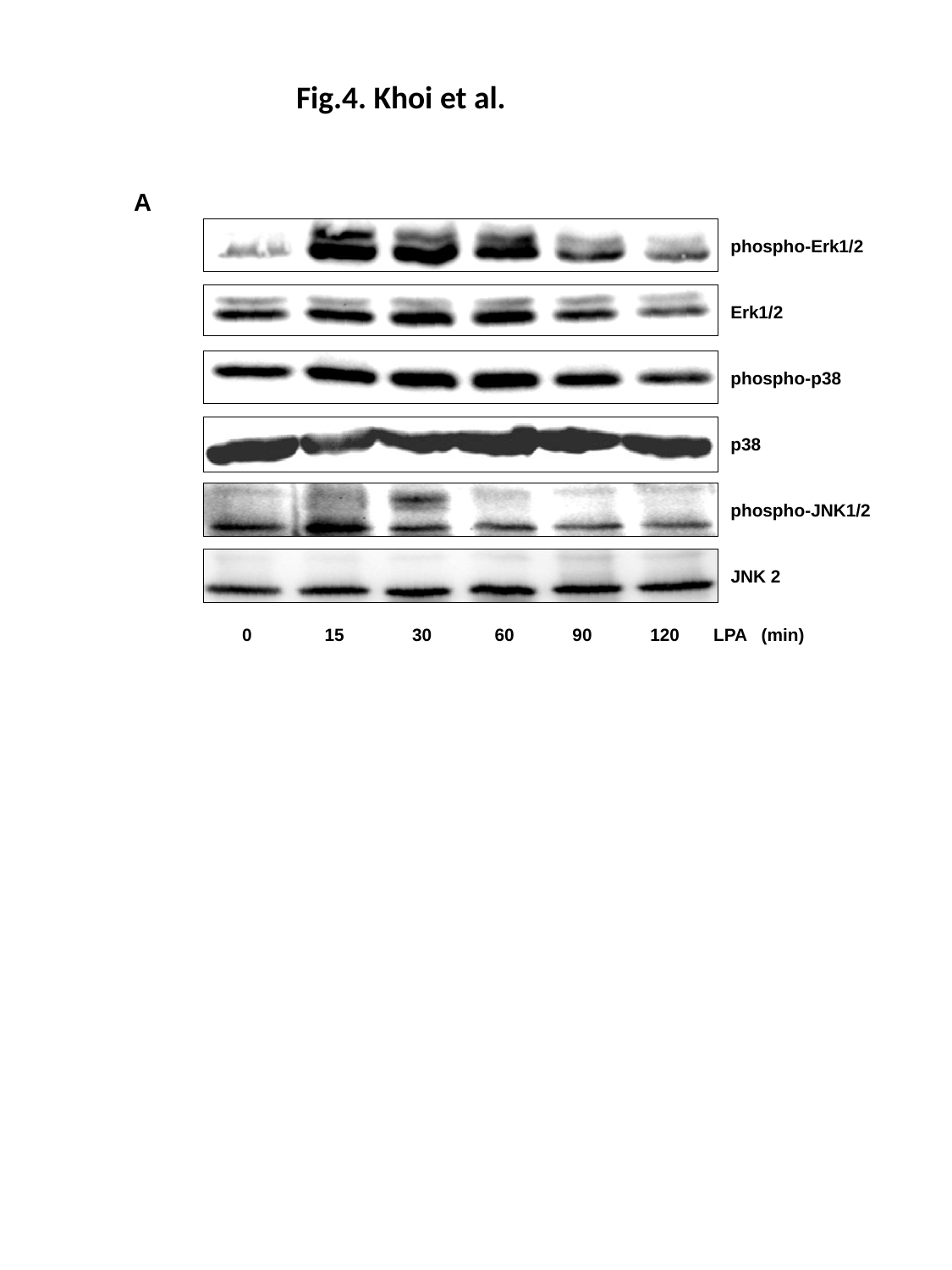

Fig.4. Khoi et al.
A
phospho-Erk1/2
Erk1/2
phospho-p38
p38
phospho-JNK1/2
JNK 2
 0 15 30 60 90 120 LPA (min)

## Slide 3
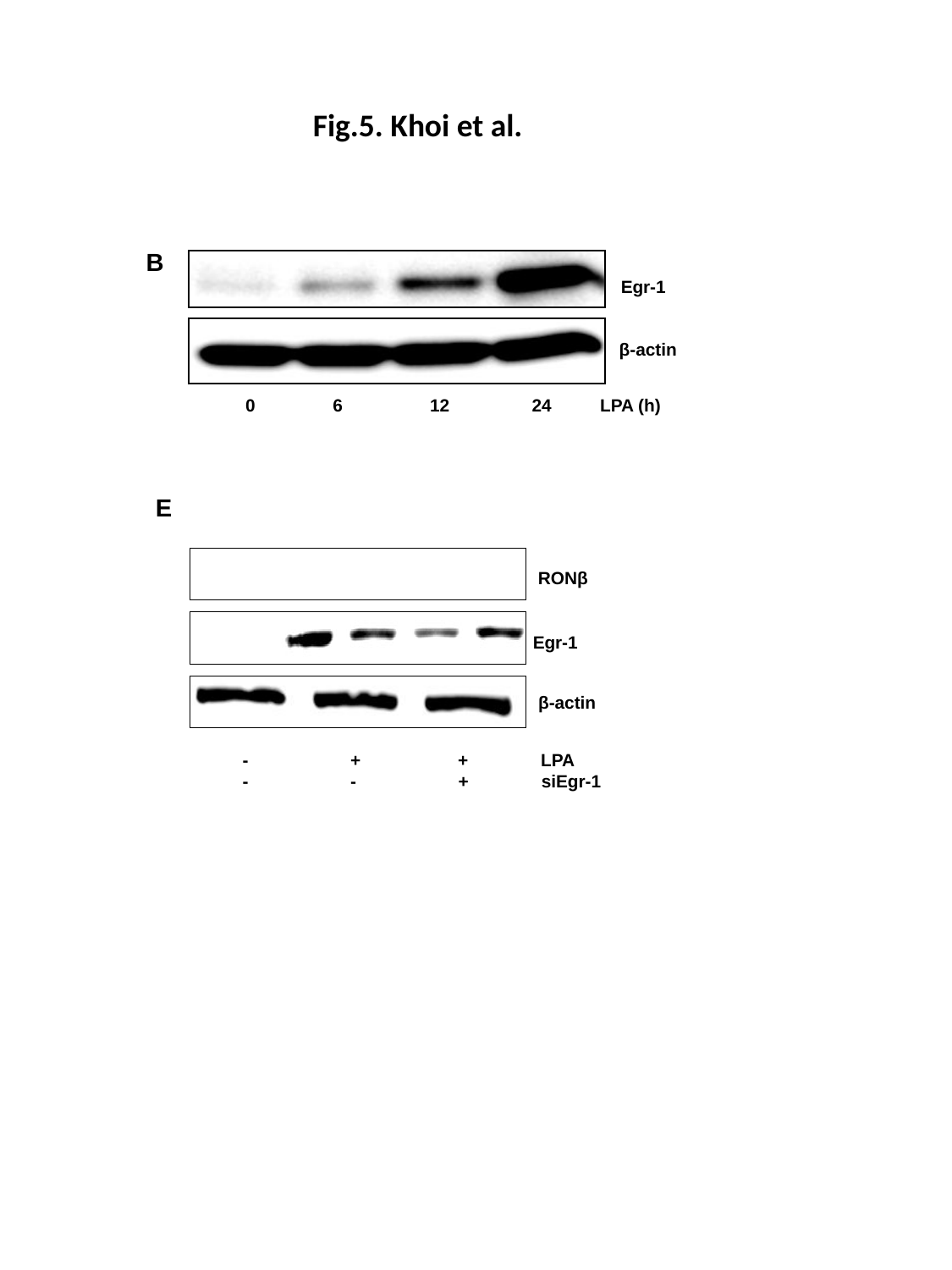

Fig.5. Khoi et al.
B
Egr-1
β-actin
 0 6 12 24 LPA (h)
E
RONβ
Egr-1
β-actin
 - + + LPA
 - - + siEgr-1

## Slide 4
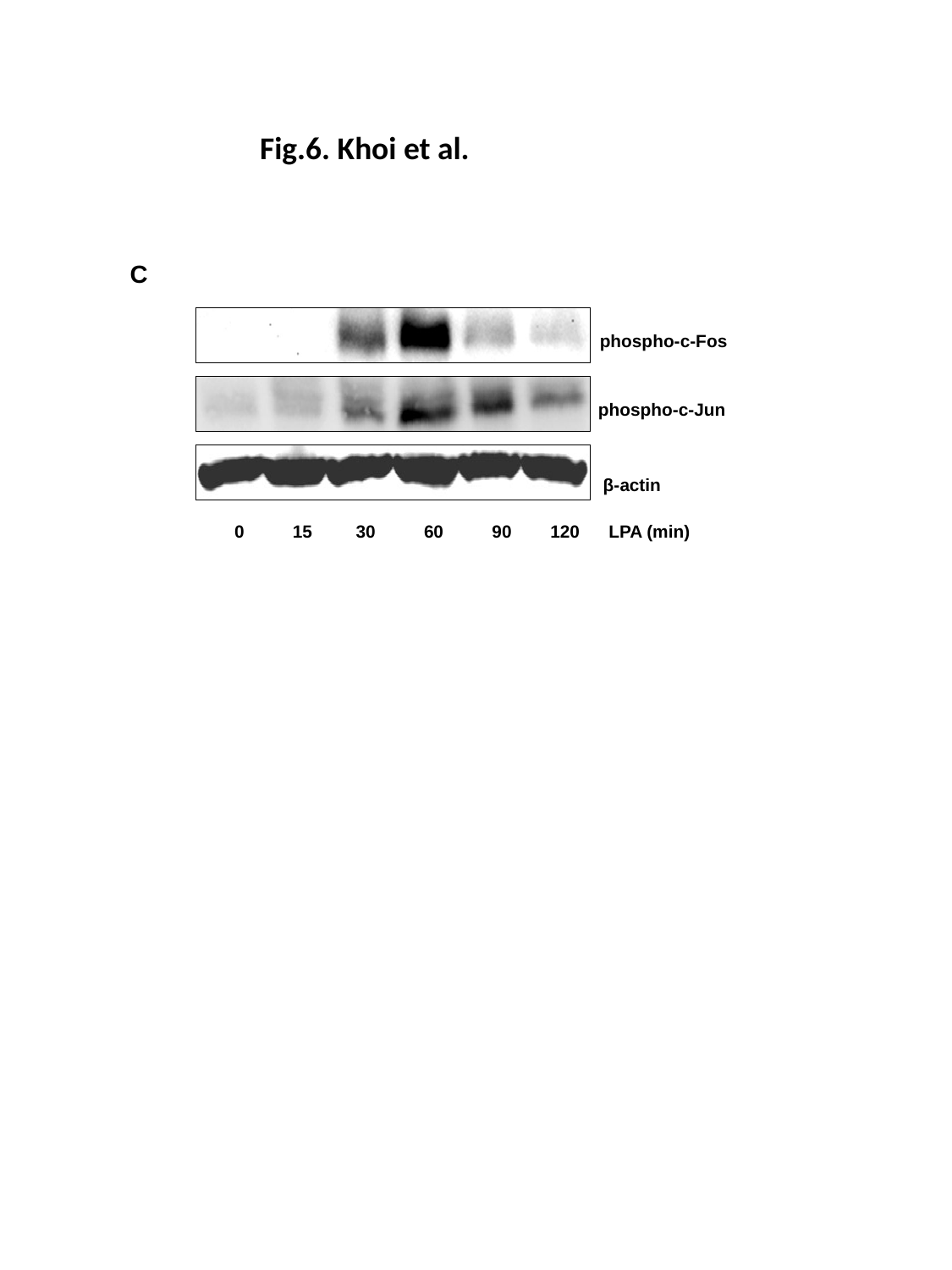

Fig.6. Khoi et al.
C
phospho-c-Fos
phospho-c-Jun
β-actin
 0 15 30 60 90 120 LPA (min)

## Slide 5
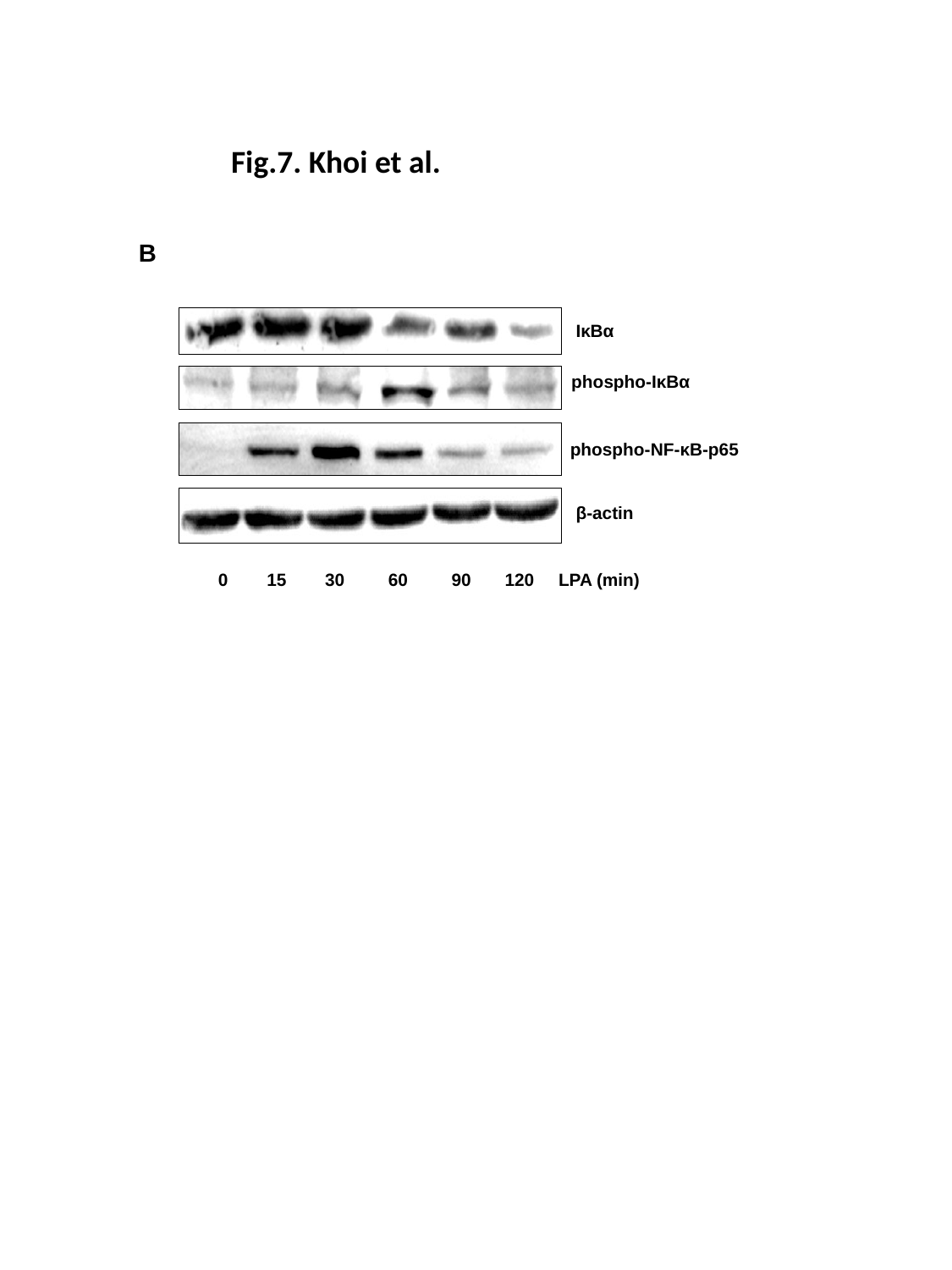

Fig.7. Khoi et al.
B
IκBα
phospho-IκBα
phospho-NF-κB-p65
β-actin
 0 15 30 60 90 120 LPA (min)
